# Giving Voice to Patients: Developing a Discussion Method to Involve Patients in Translational Research

**DOI:** 10.1007/s11569-018-0319-8

**Published:** 2018-07-09

**Authors:** Marianne Boenink, Lieke van der Scheer, Elisa Garcia, Simone van der Burg

**Affiliations:** 1 Department of Philosophy, Faculty of BMS, University of Twente, P.O. Box 217, 7500 AE Enschede, The Netherlands; 2 Malden, The Netherlands; 3 Departments of Social Medicine and Community Genetics, Free University Medical Centre, Amsterdam, The Netherlands; 4 Wageningen University and Research, Wageningen, The Netherlands

**Keywords:** Translational research, Patient involvement, Experiential knowledge, Biomedical innovation, Research and development, Discussion method

## Abstract

Biomedical research policy in recent years has often tried to make such research more ‘translational’, aiming to facilitate the transfer of insights from research and development (R&D) to health care for the benefit of future users. Involving patients in deliberations about and design of biomedical research may increase the quality of R&D and of resulting innovations and thus contribute to translation. However, patient involvement in biomedical research is not an easy feat. This paper discusses the development of a method for involving patients in (translational) biomedical research aiming to address its main challenges.

After reviewing the potential challenges of patient involvement, we formulate three requirements for any method to meaningfully involve patients in (translational) biomedical research. It should enable patients (1) to put forward their experiential knowledge, (2) to develop a rich view of what an envisioned innovation might look like and do, and (3) to connect their experiential knowledge with the envisioned innovation. We then describe how we developed the card-based discussion method ‘Voice of patients’, and discuss to what extent the method, when used in four focus groups, satisfied these requirements. We conclude that the method is quite successful in mobilising patients’ experiential knowledge, in stimulating their imaginaries of the innovation under discussion and to some extent also in connecting these two. More work is needed to translate patients’ considerations into recommendations relevant to researchers’ activities. It also seems wise to broaden the audience for patients’ considerations to other actors working on a specific innovation.

In the last decade, claims that patients (or users more broadly) should be involved in deliberations about and design of biomedical research have become commonplace. Patient organisations, policy makers, and scientists alike argue that patient involvement is beneficial to research and innovation, even though the reasons for such claims vary [1–8]. At least in some countries, funding organisations increasingly require applicants to involve patients in (the preparation of) their project. More or less simultaneously ‘translational research’ emerged as a buzzword in the biomedical domain, indicating that dedicated efforts are required to stimulate that biomedical research leads to improved health care and quality of life [9–11]. There are good arguments to make patient involvement part of such translational efforts [12, 13]. However, since there is ample evidence that effectively involving patients in research is challenging, such involvement needs to be carefully designed. In this paper, we describe how we developed a method to involve patients in translational research that aims to circumvent well-known obstacles to and pitfalls of patient involvement.

After describing the context in which we developed this method and the steps involved, we briefly clarify how we use the terms ‘translational research’ and ‘patient involvement’ and we specify the potential added value of involving patients in making research more ‘translational’. We then discuss the potential challenges and pitfalls of meaningful patient involvement as known from the literature. This leads to the formulation of three requirements that any method to meaningfully involve patients in research aiming for translation should satisfy. In the remainder of the paper, we describe the discussion method ‘Voice of patients’ that we developed on the basis of these requirements, and briefly discuss how its first use in four focus groups worked out. We conclude with a reflection on the advantages and limitations of our method.

## Background

We developed the discussion method in the context of a research project funded by two organisations interested in how to involve patients in (specifically) translational biomedical research. To ensure our work would be rooted in real-life research practices, we collaborated with two biomedical research projects. These projects first of all functioned as case studies to explore translational research in practice, including current ideas on what enables or hinders patient involvement in such research. They also served as models for intended use: the method we developed had to be usable in the context of these and similar projects. Hence, the two projects also served as testing ground for the draft method. Both projects aimed to identify and validate molecular biomarkers. As a result, our method also aimed to facilitate patient involvement in this type of research. One project aimed to develop molecular markers (using a number of different imaging and biochemical technologies) to diagnose and stratify (subpopulations of) individuals with very early, early, and established rheumatoid arthritis with different characteristics of progression of the disease and different responses to treatment modalities. The ultimate aim was to predict disease development and therapeutic responses, enabling effective therapy selection. The second project tried to identify which genomic markers are activated in the tumour of advanced-stage cancer patients (requiring a biopsy of the metastases), the results of which were then used to select patients for inclusion in a trial with a treatment tailored to their genomic profile. So here the ultimate aim was improved stratification of patients for treatment purposes.

To develop a method to meaningfully involve patients in these and similar projects, we went through three stages; in each of which, we used a variety of sources and methods. In the *preparatory stage*, we first reviewed the literature on patient involvement in biomedical research, as well as on translational research and innovation processes. This helped us to come up with a preliminary list of requirements that any attempt to involve patients in translational research should meet. We also interviewed researchers and clinicians involved in the two translational research projects, and did individual and group interviews with patients from the potential target group of the technologies these projects aimed to develop. In these interviews, we explored patients’, researchers’, and clinicians’ thoughts about the relevance and potential problems of involving patients in translational research. This helped us to refine the list of requirements and to decide which type of method to develop. In the second, *development stage*, we determined the actual content of the discussion method. We analysed the literature on ethical and social issues of biomarker-based health care. Focusing on the two research projects mentioned above, we analysed the project documents to identify their aims and expectations, as well as the implied images of the future. We observed current medical practices the projects aimed to innovate, to explore how they related to the envisioned innovations and attended research meetings to become more familiar with the current way researchers in the projects go about realising their aims. Semi-structured interviews with patients gave us further insight into which issues or concerns might be at stake for patients in relation to each research project. In total, we did 16 interviews with individual patients (or their parents), five group interviews with different sets of patients, five individual interviews with physician-researchers, and another five with lab technicians. Both literature and empirical findings fed into the content of the method. In the final, *evaluative stage*, we experimented with the first version of the method in four focus groups, focusing on the two translational research projects. The experiences gained helped us to further improve both the format and the content of the method.

## Translational Research and Patient Involvement

To justify why patient involvement in translational research might be useful, we first need to be clear what we mean with ‘patient involvement’ and ‘translational research’. To start with the latter, the concept of translational research (or translational medicine) is pretty recent, but already interpreted in many different ways [9, 14]. Basically, however, it is used to refer to a wide array of activities to stimulate that insights gained in biomedical research lead to novel diagnostics and treatments as quickly as possible [10, 11, 15–17]. The concept ‘translational research’ thus does not single out or stimulate a new type of research; rather, it focuses attention on how to make research results more translatable. It invites thinking about how best to connect different stages of the research and development (R&D) chain to foster research-based innovations of health care practice that benefit patients, caregivers, and/or society. A wide range of measures has been proposed to further such translation, like setting up large-scale R&D collaborations between academia and industry, strengthening clinical experimental platforms, training and support of dedicated personnel, and improving the coordination of research teams [14]. Surprisingly, however, involving patients is hardly mentioned as a means to further translation.

Patient involvement, like translational research, can have different meanings and take many forms. We use ‘patient involvement in research’ to denote all activities aiming to open up patients’ experiences and values for consideration and inclusion in scientific research [5, 18–20]. Such involvement can be promoted for normative and/or practical reasons. It is thought to enhance the democratic legitimacy of research and the resulting innovations [1, 6]. Moreover, such involvement may lead to better research and better innovation, because patients know best what is important to them and what might cause problems for them [1, 21]. An important assumption underlying practical arguments for patient involvement in research is that the life experience of patients leads to ‘experiential knowledge’, and that this knowledge may complement the expertise of researchers [22].This experiential knowledge should not be framed too narrow. The experience of patients is neither confined to health care, nor is it ‘just subjective’. First of all, patients have jobs, families, various interests, worries, and desires. The distinction between medical and other matters is often irrelevant to patients [23]. Moreover, inviting patients to exchange their experiences may lead to insights that go beyond the subjective level [5]. The point of involving patients in research is, then, to open up the experiential knowledge of patients and to explore where this knowledge should and how it could be taken into account when developing novel biomedical technologies.

If we understand translational research and patient involvement in this way, in particular the practical arguments to involve patients seem to be very relevant to the aim of enhancing ‘translation’ in research, regardless of whether this research is explicitly labelled ‘translational’ or not. If the aim is to realise innovations in health care that benefit patients, involving patients may help to shape innovations in such a way that they are indeed considered beneficial by this group of end users. Such involvement should not, moreover, be postponed until the aimed for innovation is available. To prevent controversies or misfits later on, it makes sense to start involving patients (and other end users) early on. Using the experiential knowledge of patients during each stage of the translational process can make the resulting innovation more *relevant* and more *usable* for patients, and it could *enhance the impact it has on patients’ quality of life in a positive way* [12, 13], as summarised in Table 1.

**Table 1:** Dimensions of innovation assessment that could profit from patient involvement  (source: [24])

Dimension of assessment	Type of questions patients may help to answer
*Relevance*	What are the needs of patients? How to prioritise these needs? Is the envisaged innovation likely to address important need(s)?
*Usability*	How are patients supposed to adjust their behaviour when using the innovation? Which burdens and risks does the innovation pose to patients? Are these adjustments, burden, and risks acceptable to patients? If not, how could they be reduced?
*Impact on quality of life*	How might the envisioned innovation interfere with patients’ daily lives? Which societal and cultural impacts (on roles, responsibilities, relations, values, identities, etc.) are anticipated? Are these impacts acceptable/desirable and if not, how could they be countered?

## Challenges to Patient Involvement

There are definitely success stories showing how patient involvement may change the direction and/or setup of biomedical research [6, 8, 18]. Moreover, funding organisations in several countries have made patient involvement part of their funding requirements, with the UK in particular being very active (see for example http://www.invo.org.uk/; or https://www.zonmw.nl/nl/over-zonmw/participatie/aan-de-slag-werkwijzer-voor-aanvragers/). However, scientific reports on past experiences with patient involvement also show that there are many challenges for successful involvement, leading to situations where patients and/or biomedical researchers feel that either the exchange itself was not very fruitful, or it did not contribute much to the innovation process. Therefore, acknowledging and dealing with these challenges is crucial if we want to avoid a situation in which obligatory patient involvement leads to ‘tick-boxing’ and superficial, useless forms of engagement.

The scientific literature points out several major challenges. First of all, time and money available are often insufficient to involve an adequate number of patients long enough to achieve a meaningful exchange between patients and researchers [6, 25, 26]. A second set of problems hampering effective patient involvement relates to the asymmetries in knowledge, language, and power between researchers (biomedical or otherwise) and patients. Scientific expertise is usually rated higher than lay knowledge [27, 28]; lay knowledge does not even fit with common ideas of *knowledge* [22, 29]. As a result, both scientists and patients frequently presuppose that patients first need to be educated before any meaningful communication about research is possible [29]. The result is that patients may become ‘pseudo-professionals’, which actually goes against the major reason to involve patients in research: to harvest and use their experiential knowledge [30]. Meaningful patient involvement requires, therefore, that the difference between scientific/clinical expertise and patients’ experiential knowledge is acknowledged and made productive, instead of erased [23]. How easy or difficult this will be depends, at least in part, on the cultural hierarchies between ‘experts’ and ‘lay people’ prevalent in a specific context, as well as on personal characteristics of the experts and the lay people involved.

Involving patients during *translational* research actually faces a third set of challenges, regarding timing and input. When biomedical research is managed to further ‘translation’, this is often guided by a pipeline model of innovation, with clear moments to decide whether to proceed to the next stage [9]. Although it is in principle possible to revisit earlier stages, the whole idea guiding pleas for ‘translational research’ is that the process should be as efficient as possible. This implies that stakeholders, including patients, had better be involved from early stages onwards, at least from the moment ideas for specific applications have been formulated. In innovation studies, it is also widely acknowledged that early (‘upstream’) stakeholder involvement is important because new technologies in subsequent stages tend to become more entrenched and thus harder to change [31, 32]. However, the drawback of such early involvement is that stakeholders are invited to reflect on an innovative technology that does not exist yet, making the topic of discussion rather vague. To facilitate meaningful reflection and discussion, then, the imagination of the patients involved has to be triggered: imagining what the technology actually will be able to do, how it will be used, and which broader impacts it may have [33–35]. Here, patients’ experiential knowledge could have added value, supplementing and enriching researchers’ visions of the future with imaginaries of what it would be like to use and live with new biomedical technologies.

A related, additional challenge that we became aware of in our preliminary interviews is that patients may have difficulty to connect stories about future innovations to their own experiences. Biomedical visions of innovation are often framed in a rather general way (like ‘we aim to develop a biomarker test that helps predict who will or will not profit from a certain drug’). On such an abstract level, these visions seemed to patients an offer one cannot refuse. They also found direct questions about these visions (‘how would such a test affect your life?’) hard to answer. However, when we asked questions about their current daily experiences of living with their disease, this usually did trigger responses relevant to the aimed for innovation. Thus, when involving patients in translational research, one should not assume patients can simply apply their pre-existing views and values to the research; rather, such views and values have to be *made relevant to* the research by and during the involvement. More specifically, in order to enable patients to use their experiential knowledge for reflection on the potential impacts an innovation could have on their lives, patients’ imagination of the future may need enhancement. Obviously, such an endeavour raises issues of who is actually constructing patients’ voices (and how), which we will return to later on.

To sum up, both the existing scientific literature and our interviews with researchers and patients indicate that there are serious, structural challenges that may hamper meaningful and effective patient involvement in research. These include power differences between researchers and patients; the discrepancy between the general nature of scientific knowledge and the personal nature of experiential knowledge; the uncertain, highly imaginary character of the envisioned innovations; and patients’ difficulties to imagine the ways in which future innovations may impact their lives. They are *structural* in the sense that they are to some extent part and parcel of the problem situation in which patient involvement is called for. For that same reason, they are also real *challenges*, which are unlikely to ever fully disappear. The extent and the way in which these challenges actually hamper patient involvement currently seems to largely depend on context specific factors, like the personal characteristics of the individuals involved. In view of the increasing calls for and expectations of patient involvement in biomedical research (whether or not translational), it is crucial to develop methods that more systematically acknowledge these challenges, and thus to help improve the conditions for meaningful involvement.

## Towards Meaningful Patient Involvement in Translational R&D

In view of both the potential and the described challenges of involving of patients in (translational) research, we set out to develop a method that first of all tackled the challenges related to the *content* of patients’ deliberations. Reasoning that patient involvement can only have added value for innovation if it succeeds in connecting patients’ experiences with innovations, we formulated three requirements. The method had to enable patients (1) to put forward their experiential knowledge, (2) to develop a rich view of what an envisioned innovation might look like and do, and (3) to connect their experiential knowledge with the envisioned innovation. We thus focused on improving the quality of the insights produced in patient deliberations, as this is a first prerequisite for uptake of such insights by researchers. Creating conditions to enable and stimulate uptake of these insights definitely requires further work, but is beyond the scope of this article.

With regard to the *first requirement* (enabling patients to put forward experiential knowledge), we concluded that if opening up patients’ experiential knowledge is the aim, it is important that patients are engaged in conversation in an open atmosphere; they should feel at ease to share anything they want, in their own way. This means they should not be required to have prior scientific knowledge (in general or about the project under scrutiny), nor be expected to speak in scientific language. Moreover, although the agenda for such meetings is delineated to some extent by the medical research the patients are invited to reflect on, it should be sufficiently open. Patients should feel invited to bring up what they think is relevant to this research. It is particularly important to make sure that the way information about the research project is presented does not overly shape patients’ responses.

At the same time, however, some structuring of the discussions by a facilitator or moderator will be necessary to enable meaningful exchange, to make sure the conversation does not focus too quickly on one aspect, and to ultimately formulate conclusions that are clear and sufficiently shared among participants. This moderator can be a humanities scholar social scientist, but also a biomedical scientist or a patient representative, provided she/he has the necessary listening, clarification, and dialogic skills and explicitly tries to reduce the influence of his/her own framing to a minimum. Clearly, creating conditions for an open conversation does not imply the setup for patient involvement will be ‘neutral’. The setting of the engagement (in the context of a specific biomedical research project), the choice for a group discussion format, and the role of the moderator all will have an influence on what will be put forward by patient participants as well. The point is to facilitate a broader and more open discussion than just bringing together patients and researchers would usually allow.

To make sure patient involvement satisfies the *second requirement* (developing a rich view of what an envisioned innovation may be like and do), it is important that patients are introduced to and can discuss the aimed for innovation in concrete terms. Even when an innovation still is ‘in the making’, patients should be enabled to imagine what it would be like to work and live with the innovation. Here again, as Bruce also notes [36], some framing of the innovation under discussion is inevitable. When providing information about the research under consideration, the future imaginaries of the scientists involved may provide a useful starting point. However, since these are often rather abstract (‘to personalize treatment’) as well as focus on health impacts only, patients should be stimulated to imagine what other impacts the aimed for innovation might have, and what the innovation might mean for different types of people involved. Using narratives about the future can be helpful here, in written or audio-visual form. For example, scenarios or vignettes imagining a wide range of potential impacts can invite patients to think beyond the intended effects of an innovation and its potential risks for health and safety [37–39]. However, if the scenarios are too detailed, participants’ imagination may be guided too much in a specific direction, so a careful balance between stimulating imagination and openness should be strived for.

The *third requirement* (enabling patients to connect their experiential knowledge with the envisioned innovation) implies that patients while imagining possible futures should be stimulated to connect their experiential knowledge with those futures. They should be invited to ask: ‘What would it mean for me if such an innovation was available? How might it affect my daily life and to what extent would that contribute to my quality of life and to a good society?’ This means that the visions of future practices should not only be sufficiently rich and varied, they should also invite reflection on the level of patients’ daily functioning. Both the information about the research project provided as input for the discussion and the moderator should fare a midway between asking very open questions that are hard to respond to, and proposing very detailed scenarios that prevent patients to come up with their own experiences. Also, collective brainstorming and exchange about potential impacts seem more helpful than asking about individual experiences only. Hearing each other’s responses may trigger less obvious thoughts and ideas. Such collective exchange may also help to assess the plausibility and desirability of certain ways of using an innovation and of specific impacts.

Reviewing existing methods to involve patients or stakeholders in R&D with the above considerations in mind [24], we decided that *group meetings* using *tools to trigger patients’ imagination* of emerging innovations seem most promising for meaningful involvement of patients in translational research. Moreover, we assigned a *substantial role to a moderator*. This person not only facilitates group discussions, but she/he also helps prepare the material serving as input for the discussion, and may subsequently communicate patients’ views to the researchers (in case these are not present). Regarding the tools to stimulate patients’ imagination, we decided against using scenarios or vignettes, because these provide images of the future that leave limited room for patients’ own imagination. Instead, we opted for using discussion cards, a concept (initially called ‘Democs’, DEliberative Meetings Of Citizens, and later also PlayDecide, see www.playdecide.eu) developed in 2000–2001 by Perry Walker at the New Economics Foundation, which since then has continued to be used in the context of citizen deliberations about new and emerging technologies [36, 40–42]. Card sets can cover a wide range of topics and issues and therefore offer rich input to enhance imagination and reflection in a structured way, but also offer participants the possibility to pick and choose the cards that they consider most relevant, interesting, or off-putting, or to add extra topics to the cards. There is therefore plenty of opportunity for participants to steer the discussion. Cards facilitate a substantive, systematic, structured, and at the same time open exchange.

A cards method called IMAGINE, developed on the basis of PlayDecide by Felt and colleagues [43] to engage Austrian citizens in debates on nanotechnology and society, seemed particularly useful for our purposes. This method includes a set of discussion cards and a concomitant ‘choreography’ for using these cards in focus groups with citizens. Even though the setting for which IMAGINE was developed (citizen deliberation on a broad set of emerging technologies) clearly differs from ours (involving patients in translational research projects), we share many of the considerations that underlie this particular method. We particularly appreciate the way the IMAGINE cards may help to stimulate participants’ imagination of a topic they might not have given much thought before, while preventing a limited ‘scientistic’ framing of the discussion. We decided, therefore, to use the IMAGINE-format as a basis for our method to involve patients in translational research projects. At the same time, we kept the requirements we formulated in mind to make sure our method would address the potential obstacles mentioned above.

## Developing ‘Voice of Patients’

As indicated above, we set out to design a method that could be used in the context of any research project on molecular biomarkers for medical use, regardless the specific type of markers, technologies, function, or disease involved, thus faring a midway between focusing on a specific research project and focusing on any kind of biomedical research. The method we developed (called ‘Voice of patients’) centres on focus group meetings of patients, facilitated by a moderator who structures the discussion in four rounds, each using a particular set of cards. The first round uses *story cards*, describing the first person experiences of a specific type of actor dealing with biomarker technology (like a researcher, physician, citizen, or policy maker). This is to draw participants into the topic without committing them to a particular perspective. The second round features *application cards*, which describe different applications biomarker technology might have, including promises, possible benefits, and potential risks. The *issue cards* used in the third round refer to ethical, economic, legal, political, and social aspects of the technology as experienced by individual users. The fourth round uses *society cards*, inviting participants to broaden their scope and reflect on the way biomarker technology might impact society at large. Although the topics of the last two sets obviously interrelate, we wanted to ensure that discussions would go beyond considerations about individual lives and explicitly stimulate participants to reflect not only from a patient’s, but also from a citizen’s role.

We based the content of the *story* and *application cards* primarily on the interviews we had with researchers in the two translational research projects we collaborated with, on the analysis of their research protocols and on our literature review of general aims of and expectations in biomarker research. The content of the *issue cards* was based primarily on our semi-structured interviews with individual and groups of patients, as well as on the literature on ethical and social issues in biomarker research and visions of biomarker-based healthcare. The content of the *society cards*, finally, was primarily based on literature research, as these topics were mentioned less often in interviews with patients and researchers. After experimenting with draft cards in four focus groups, we developed a fully revised set of cards and a guideline for using these [44].

A focus group ideally consists of six to ten patients, a moderator, and an observer taking notes. The meetings start with a short introduction (of about five minutes) of the research project under discussion. This can be a video or a live presentation, prepared by the organizers of the patient involvement in collaboration with the biomedical researchers. In case of a live presentation, this may be done by a biomedical scientist or the moderator. The actual content should include a description of the actual research (what is investigated, how and why), as well as the envisioned future product and user practice. Participants may ask questions for clarification.

**Fig. 1:**
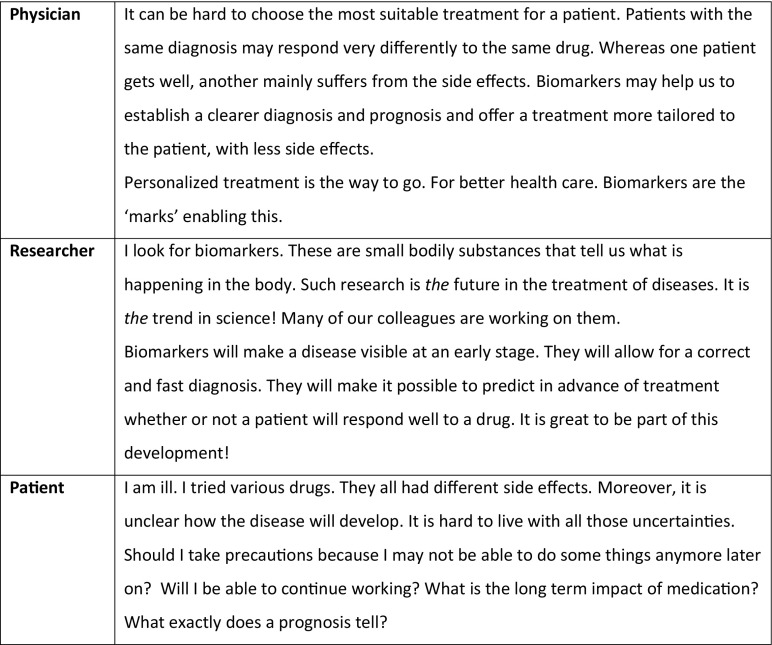
Examples of story cards

Then, the moderator introduces the *story cards*. Six cards tell stories about the research from the perspective of a researcher, a patient, a physician, an entrepreneur, an ethicist, and a politician. Figure 1 shows a few examples. The participants are all asked to read the cards and choose one. They are not given any criterion to base their choice on, but are asked why they chose a particular card. The point is that not only the card chosen, but also the reasons for choosing them may reveal something about participants’ interests and motives. This round lasts about fifteen minutes. The main point is that participants are introduced to the research from a variety of perspectives.

**Fig. 2:**
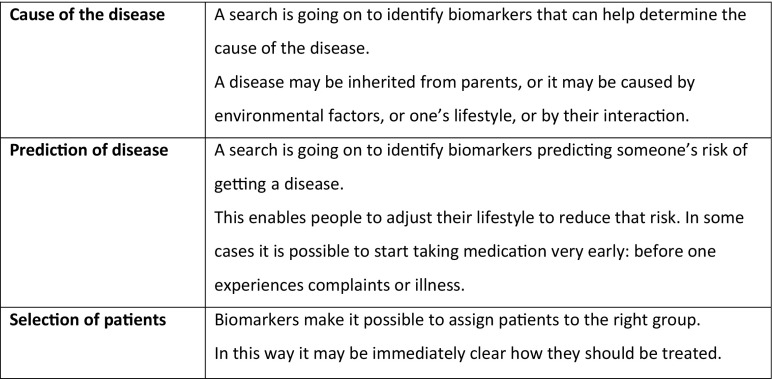
Examples of application cards

The participants are then asked to read the *application cards* (see Fig. 2 for some examples). All cards describe in a concise and schematic way potential applications of biomarkers, no matter whether these are almost realised or as yet rather speculative. The description is not tailored to the project under discussion, but phrased in general terms, inviting participants to actively search for the connection with the project presented earlier. We developed nine cards for the different functions biomarkers might fulfil. Depending on the aims of the specific research project under discussion, cards can be in-/excluded or even added, if necessary. The moderator decides about this in consultation with the biomedical researchers, in advance of the meeting. After distributing the cards, the moderator asks all participants to select two cards (again, without giving criteria). Participants explain their choice, which leads to an exchange on what is relevant to patients and why. The aim of this round is to help participants form an idea of the different types of applications that might result from the research, and to invite first, open reflections on (for example) the benefits, drawbacks, possibilities, and/or limitations of these applications. This can result in feedback on the relevance of these applications for patients, as well as on problems and risks they perceive. This round also takes about fifteen minutes.

**Fig. 3:**
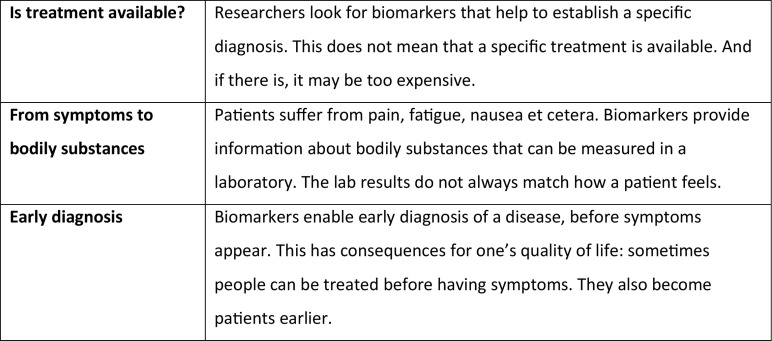
Examples of issue cards

In the third round, the *issue cards* confront participants with a wide range of topics and questions, inviting reflection on the potential consequences of the aimed for technology on the life of patients (for examples see Fig. 3). Again, these texts are not specific to the project under discussion; patients are expected to link the general texts to the discussed research themselves. We developed 26 cards, showing the diversity of issues that may arise; the moderator together with the researchers can decide in advance of a meeting to exclude cards irrelevant to the project under discussion. After reading, all participants choose three cards and explain their choice. They can also write their own card, adding a topic to the discussion. Choosing three cards makes it possible to construct complex arguments. The resulting discussion may help participants to develop and/or deepen their position on these issues and to formulate concerns they would like to see taken into account during the innovation process. These considerations may pertain to the relevance and usability of the envisioned innovation, as well as to the impact it may have on the quality of their lives. The round takes about thirty minutes.

**Fig. 4:**
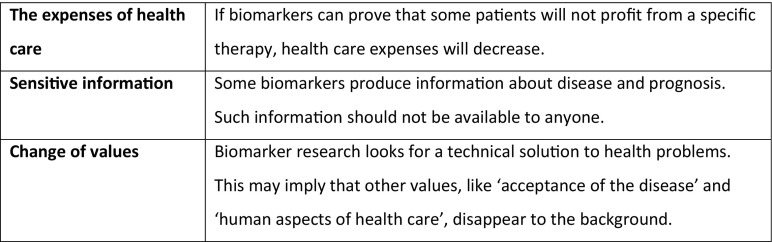
Examples of society cards

In the fourth round, participants reflect on the potential impact of the research on society and culture. They read the *society cards* and choose two (or add one). These cards (eight in total) describe how biomarker research may for example transform personal identities, the way different groups interact with each other, or how goods are distributed in society. The emphasis is on indirect, unintended, and often morally ambiguous impacts of using a technology (for examples see Fig. 4). The choice requires patients to relate their experience and interests as a patient to their role as a citizen, deliberating how innovations that are personally relevant to them may work out on a collective level. The aim is to explore how acceptable social impacts are to patients; this round may actually change their earlier views on the relevance of the research. The round lasts for about fifteen minutes.

To wrap up the focus group meeting, all participants are asked, one by one, what they think is the most important thing to convey to the researchers.

## First Experiences

We tried out the first version of *Voice of patients* in four focus groups, two for each of the two research projects described above. Since we felt that the presence of a biomedical researcher has both advantages and drawbacks, we experimented with both formats. A biomedical researcher of the project under discussion took care of the kick-off presentation and was present in half of the groups, with the explicit instruction to act as an observer only after the kick-off. In the other half, the moderator took care of the kick-off presentation and no biomedical researcher was present. All groups were moderated by one of the authors (EG or LvdS), with the other of these two present making notes and as an extra pair of hands and eyes. A summary of the main characteristics of the groups can be found in Table 2.

**Table 2:** Overview of focus group characteristics

Group no.	Project under discussion	Number of participants	Biomedical researcher present?	Introductory presentation of research project by
*Group 1*	Genomic tumour marker for treatment stratification	11	No	Moderator
*Group 2*	Biomarkers for improved diagnosis and treatment stratification of RA	8	No (representative of funder present as observer)	Moderator
*Group 3*	Genomic tumour marker for treatment stratification	7	Yes	Biomedical researcher
*Group 4*	Biomarkers for improved diagnosis and treatment stratification of RA	7	Yes	Biomedical researcher

Based on the experiences and feedback received from participants and observers during these pilots, the initial cards set and the accompanying instructions were finalised and made publicly available [44]. We do not have the space to extensively discuss results here, but let us highlight some first experiences and reflections shared by participants in evaluative discussions at the end of our focus groups.

As for the content of the discussions, it is safe to say that in all four groups, the format and the cards led to lively discussions on biomarker research and on the experiences and lives of patients with either cancer or RA. Patients were very enthusiastic about the conversations and indicated that they felt triggered to reflect on issues beyond what they would have individually thought of. They also gave some very practical feedback on the method. For example, the phrasing of the text on some cards was not fully clear. It also turned out that cards with statements elicited better responses than cards with questions. If a card posed a question, participants often started answering that question, instead of reflecting on how the topic of the card could be relevant to the project under discussion. In response, the text on the cards was clarified where necessary, and we reformulated text phrased as questions into statements. Furthermore, some participants found the high number of cards used in the third round at first sight a bit daunting. In practice, they managed dealing with them quite well, and the high number of cards is important to trigger participants’ imagination in diverse ways. We therefore included some considerations in the users’ guide on whether and how to select cards.

As for the role of the biomedical researchers, when they were present in the groups patients seemed to feel sufficiently comfortable to share their experiences. An obvious advantage was also that researchers present got a first-hand view of patients’ considerations. However, we also noted that some patients were prone to start asking questions to the researcher as an authority on the research under discussion, thus potentially giving more weight to the scientific perspective, and that it was challenging for some of the researchers to stick to the listening mode. So we actually re-experienced some of the advantages as well as the drawbacks of having a biomedical researcher present in the group, suggesting there is not one model that is clearly desirable in all cases.

Apart from these practical considerations, the main question of course is how successful the method was in realising the three conditions formulated above. Did the method enable patients

1. to put forward their experiential knowledge;
2. to develop a rich view of what an envisioned innovation might look like and do; and
3. to connect their experiential knowledge with the envisioned innovation?

### Inviting Experiential Knowledge

The cards method both in setup and content aimed to keep in check the influence of biomedical expertise and framings and to invite the shaping and sharing of diverse patient views on emerging biomedical innovations. In the focus groups we organised, a completely biomedical framing of the discussions was indeed avoided. This is illustrated by the following extracts from focus group 4 (on RA, with a researcher present). The first three participants who spoke up in round 1 all chose the card with the researcher’s story. But they did this for different reasons: the first felt biomarker research might ‘help prevent fear and suffering for a large group of people’, the second stated that ‘research is the first step to know more, which is good to be able to act more carefully’, and the third one added that ‘science shows what is going on’. These positive attitudes towards science were, however, immediately discussed more critically when the last speaker added: ‘Anyway, there will of course also be a group whose PET-scan does not show anything and who feel not understood… That is the other side…’, apparently taking into account that biomarker research probably will not be a complete success and that failure has an impact on patients as well. At this point, the biomedical researcher intervened, stressing the positive impact of future biomarker technologies: ‘Yes, but then… it is, of course, always the case that you identify at least those people, you are certainly able to identify a large group of people, that is the basis…’ Another participant replied, however, that one should also be aware of false diagnoses. A brief discussion ensued, leading to the observation that ‘biomarkers do not tell everything and you [i.c. physicians] should think hard and deep about everything you do’ (all citations from focus group 4). As this sequence of contributions shows, even while the biomedical researcher intervened and highlighted the positive contribution of biomarker technologies, participants were quite capable of identifying and acknowledging ambivalences and complexities, based on the plurality of perspectives and storylines the cards in round 1 provided them with.

Furthermore, the cards method did help participants to express their experiential knowledge. While the cards offered concrete input, the participants were free to select the cards that somehow interested them. When asked to give reasons for their selection, participants often referred to their own experiences and also made claims about patients’ experience in general. An example is the exchange that followed the selection of the issues card ‘Living with a chronic disease’ (in round 3 of focus group 1). The card read: ‘Many diseases cannot be cured, but life expectation can be improved. This means that patients live longer with a chronic disease. What does this mean for the patient’s life?’ The person who chose this card explained:

V: Well, I learnt that people adjust their standards when something awful happens to them. (..) I feel that I did not adjust my standards yet. Rather dead than in a wheelchair - but once I need a wheelchair I may not think that anymore.

E: You adjust your goals. (….) Maybe the cancer hardly bothers you, but at the same time, the consequences of the treatment may last and that is an impact of chronic, of treatment anyway. (…) You know that there still is something in your body that might reactivate and that, has, does impact your attitude towards life and how you feel. So I think that if more cancer types become chronic, it becomes more important to investigate other things than the cancer itself, to find out what its impact is and how to deal with that and improve, try to support.

(…) For example, yes, I had colon cancer myself and that is fully cured, but I still have a colostomy left, yes, that’s something you can live with quite well, but it does have quite an influence, and there are many things like that, which one could investigate and support’. (focus group 1)

Triggered by the card, the participants jointly explore what it could mean to live with cancer as a chronic disease. Their personal experience is used when they imagine adjusting standards for what is an acceptable quality of life, or referring to needs for support that might remain, even if the disease itself would be tackled.

Another example of how experiential knowledge was brought forward in the focus groups is how a card on the application of biomarkers for early diagnostics led to reflections on the drawbacks of early treatment in group 4 (on RA). A participant told that her mother died of the side effects of RA medication, which made her reluctant to start with medication before her 50th birthday. Both examples show how participants’ experience led them to qualify the positive framing of research aims like ‘turning cancer into a chronic disease’ or ‘early diagnosis enables early treatment’.

### Developing a Rich Imagination of the Aimed for Innovation

The second aim of the cards method is to help participants develop a rich imagination of what the innovation would be like or do. Especially, the second round of the conversation, structured by the application cards, was helpful to realise this. After the kick-off presentation of the research project under discussion focused on its scientific aims and the story cards in round 1 helped to broaden the number of perspectives taken by the participants, the application cards in the second round helped to broaden participants’ view on the future(s) of biomarker research. The wide range of aims presented on the cards triggered reflection and discussion on what would be the most valuable aims of biomarker research in general. They helped put the aims of the project under discussion in perspective and invited reflection on the positive potential, as well as the possible drawbacks of biomarker research in the domain under discussion. In focus group 2 (on RA), this led, for example, to the observation that prediction and early diagnosis of disease are ‘too dangerous’. Being informed that you have RA while you do not experience any symptoms raises dilemmas that several participants would rather avoid, for example about whether or not to inform your insurance company or bank. Moreover, several participants argued that they would have lacked the motivation to submit themselves to a PET scan before they got complaints, as in the following excerpt:

A: I was 50 when I first got RA, and I was an entrepreneur at the time. Work overwhelmed me, I was very stressed, in hindsight I think my RA may have been caused by that stress as well. (..) But I would not have gone in, not during my lifetime. I did not have time for that at all, come on! (…)There’s nothing wrong with you, isn’t it? You are not going to lie down in there if there’s nothing wrong with you, are you?

(…)

F: Yes, but what you just said, then I thought, yes, I would go after all. Because I think, for example, my sister who is 1 year younger than I am, she recently called me: ‘My hands are causing trouble, and it started with you in that way.’ We had quite a long talk about that, and I can’t avoid thinking of that now. (..) If that would happen now, I would say: go for it, you.

B: Here it is more genetically determined.

(…)

G: But that is, indeed, if you do not have someone in your environment, than you would not go here anytime soon. I also certainly would not have done that, in such a case I’m thinking, oh, well… (focus group 2)

In the other group focusing on RA (group 4), similar discussions took place. These ultimately led to the conclusion that as long as options for early intervention and prevention are lacking, developing markers to *monitor* patients would be a much stronger argument in favour of biomarker research than identifying markers for early diagnostics, because of the ethical issues the latter raises. PET scans were therefore more appreciated as monitoring devices rather than diagnostic ones. In group 3 (on cancer), a similar shift was proposed when one of the participants argued that the selection of patients enabled by biomarkers was important, not to select patients who are likely to profit from treatment, but even more to deselect those who would only suffer from side effects. The conversations in the diverse focus groups thus sometimes led to a broadening of the goals research should aim for, or even to proposals to apply the technology for different goals.

The third and fourth rounds of the conversations (about impacts on the lives of patients and societal impacts) invited an even broader reflection on the future of the developing biomarker technology. Topics discussed included the following:

- What are relevant phenomena to study in the research project? Some participants argued that ‘health gains’ were often interpreted in a narrow way; in the case of RA, fatigue was put forward as equally important to monitor and study as ‘disease activity scores’. In one of the groups on cancer (group 3), there was extensive discussion on what constitutes a ‘good diagnosis’, with participants reflecting on the relative importance of unambiguousness, certainty, and the possibility to take action.
- Participants in several groups stressed the importance of having a holistic view of the patient as a human being, instead of focusing on the diseased body only. It was also suggested that there should be a clear ‘feedback loop’ between patients participating as research subjects in a project and the researchers, so that unintended and unexpected consequences of the biomarker technology on human lives can be taken seriously.
- Participants indicated that they would appreciate ‘honest’, realistic information about a research project, avoiding huge claims like getting rid of cancer or making cancer chronic. They acknowledged that research projects under discussion would not benefit participants, and that it might take more than 10 years before these projects would lead to improvements in care. Many felt participation in such research is important nonetheless, and that it would be better to tell participants that taking part in a trial means helping others, rather than suggesting it might benefit them.
- Participants realised that biomarker research usually requires storing and linking of huge data sets, and identified privacy protection as an important concern. Group 1 (on tumour markers), for example, agreed that the material and information collected should be used for research and medical purposes only, excluding access for insurance companies, employers, and banks for example).
- Participants realised that knowing biomarker test results might create new, possibly hard choices and obligations. In group 3 (on tumour markers), participants for example imagined how knowledge about the cause of the disease might have far-reaching implications for the choice whether or not to start a family and for being held responsible for one’s disease.

Overall, then, the cards method was successful in triggering participants’ imagination of the potential impact of the research project under discussion. Participants quickly went beyond the explicit objectives of and motives for the project highlighted in the kick-off presentation. This was apparently so successful that one participant in focus group 4 (on RA) ultimately reflected that the cards possibly were ‘too problematizing’, at the expense of exploring positive possibilities and suggestions.

### Connecting Experiential Knowledge with the Envisioned Innovation

The third and last aim of the cards method, connecting patients’ experiential knowledge with the envisioned innovation, proved the most challenging. On the one hand, the cards did trigger participants to voice considerations rooted in their earlier experiences as patients that were pertinent to the research project under discussion. So we consider the cards method successful in this respect. On the other hand, more seems needed. Our main argument for involving patients in translational research was that their input may help to make an innovation more relevant and useable for patients, and that it may increase its positive impact on patients’ quality of life. Our current study did not look into the ultimate impact of patient involvement on researchers’ thinking and decision-making, let alone on the resulting innovations, so we cannot evaluate whether the outcome of the innovation process was indeed affected. To enable such an impact, however, patients’ input should at least be formulated in such a way that it can be taken up by the actors involved in the innovation process, in particular by the researchers. How well did the method succeed in making this connection?

First of all, patients voiced quite some practical suggestions. These regarded, among others, communication with patients as research subjects, for example ‘Make sure that there is a continuous opportunity for patients to notify the researchers of things you notice while being part of the experimental research. So take care of good documentation and a procedure to respond fast to negative consequences’ (group 1). There were also suggestions about recruitment of research subjects: healthy subjects, but also a number of patients might not be willing to subject themselves to a PET scan for research purposes only, without clear personal benefits (group 2). Outcome measures were a point of attention as well: genes and proteins may be relevant to researchers, but these interest patients only when they are related to their subjective experiences (group 2).

Overall, patients voiced many considerations regarding the relevance of research. For RA patients, research on fatigue may be more relevant than research on biomarkers (group 4). And patients in all the groups clearly indicated they would prioritise research aiming to improve diagnosis, therapy, and monitoring above research aiming for screening and prediction. In focus group 4, this triggered the researcher who was present to check her project goal: ‘We are focusing on people who are already experiencing pain and who are looking for help. Did I understand correctly that you think this is useful?’ (final round group 4) The group confirmed this, but also discussed how even monitoring of individuals with complaints might lead to uncertainties as to when biomarker test results justify intervention (i.c. prescribing drugs), and who should decide about this.

The biomedical researchers who were present in some of the focus groups were positively surprised by the discussions and insights the cards generated. They indicated that they had heard novel considerations that they would not have thought of themselves. For quite some considerations, however, it was not very clear how, or even whether, researchers could take them into account in their own work. Here, we actually encountered several ‘translational challenges’ of our own.

The first one has to do with insufficiently specifying the implications of the insights voiced by patients for the research project under discussion. Many of the considerations put forward by participants were framed on a rather general level. Considerations about, for example, the psychological impacts of biomarker testing, the ‘chronification’ of disease, or drawbacks of screening and early diagnostics, were put forward in a way that indicated a general concern rather than a specific worry about the project at hand. This was probably partly a result of the way the cards framed topics. To make the cards useful for a variety of research projects, we presented the stories, issues, and themes in a general way. Participants are therefore supposed to make a double connection when selecting cards and reflecting on the reasons for their choice: first of all with their own experience, and secondly with the project under discussion. In practice, they did not have a lot of difficulty with the first, as the examples of their contributions cited above testify. However, explicit connections with the research project were made much less often. The moderators of the meetings explicitly invited participants to make the second connection at the end of the meeting, by asking them what they would like to feed back to the researcher. However, by then, it was hard for participants to remember all the different concerns mentioned and often there was not sufficient time left to elaborate recommendations. It seems that to stimulate translation of considerations into advice for the research project, more active and frequent summarising and probing by the moderator may be needed. Another option would be to specify the text on the cards for each of the research projects they are used for.

Two other factors seem to be at play in the only partially successful translation of patient insights into advice for the research project. First, looking at the type of considerations put forward, many were directed at the relevance of the research, rather than at its usability. Moreover, and partly related, the concerns raised often seemed to point at issues beyond the researcher’s domain of influence, thus targeting other actors involved in the innovation process. Both factors can be seen at work in the following excerpt:

B: ‘I also chose “tailored therapy”. Especially because, and I think S. already said this as well, you need to look at the person as a whole, so at his whole situation. What is his age, what is he doing, how does he live. And this is sometimes, with therapies, in my experience, is sometimes forgotten, you are indeed perceived as your, eh, disease. (…) I think this can be improved, and for me, that belongs very much to “tailored therapy”. Just like R [the researcher] said, everyone has a different form of RA. Not only on the technical side, but also in the way he deals with it and what kind of person he is. That is not something you can do a lot about. Everyone experiences it differently, deals with it differently. And I think this is something that does not get a lot of attention during treatment. Hence: tailored therapy’. (round 2, group 4)

Participant B here voices concern about the rather narrow view of ‘tailored treatment’ promoted in biomarker research (in general and in the RA project discussed in this group). He agrees every patient should be treated as unique, but not only in a ‘technical’ sense, differentiating different disease types. Differences in character and way of dealing with disease need to be accommodated as well. This remark puts the relevance of biomarker research in perspective. What this implied for research and innovation was not explicitly discussed. One could take it as a plea for less reductionist types of research, or as a plea for additional types of research beyond the biomedical. One could also surmise that implementation is crucial: ultimately, patients should be the ones deciding what type of treatment fits best with their situation (still leaving open what is needed to ensure that all patients can make a well-considered choice and get the care they need). In both interpretations, the role for the researcher is limited. Funding organisations and health care providers, rather than researchers, may be the addressees of this message.

Considerations regarding relevance often seemed to be somewhat ambivalent. Participants clearly saw the potential, but also the limits of biomarker research, both in general and of the project under discussion. At the end of the meeting, when asked whether the project should continue, participants always responded positively. At the same time, preceding discussions implied that from the participants’ perspectives, many other types of research are needed as well. It should be recognised that such considerations for researchers are hard, if not impossible, to accommodate. Funding and management systems create structures and obligations for researchers that make it difficult to deviate from what was promised and planned in current projects. Even if we think of such patient input as relevant for a researcher’s next projects, rather than for the current one, a researcher’s background and training will usually limit possibilities to change track. This means that considerations regarding relevance should be addressed not only to researchers, but also to (among others) funding organisations.

Similar questions regarding who should respond to patient input occur when patients voice considerations of usability. When for example outcome measures are the issue, researchers clearly can take this into consideration, at least when developing their next project. Concerns about the psychological impact of biomarker testing, implications for insurance, or a patient’s freedom to decide about his/her own care seem to be relevant for health care providers and policy makers, rather than for researchers. The researcher present in focus group 4 at some point observed that extensive training of GPs would be necessary if the biomarker test for RA would become available in standard care. This indicates that patient concerns may go beyond the researcher’s sphere of influence. In this respect, the organisational setting of our focus groups (linked to specific research projects and feeding back results to researchers only) may have been too limited.

Overall, then, the considerations put forward by the participants were usually rooted in their experiences as patients, and also relevant to the aimed for innovations. Translating them into advice that could be taken up by those involved in the research project under discussion was more challenging, however. More active probing by the moderator or specification of the cards may be needed, but broadening the audience with other actors involved in innovation seems also advisable.

## Discussion and Conclusion

If patients are the supposed beneficiaries of biomedical innovation, there are important normative and practical reasons to involve them in the innovation process. Such involvement fits quite well with current pleas for ‘translation’ in biomedical research, since the experiential knowledge of patients may help to realise innovations that are relevant and useful for them. However, involving patients in biomedical research is not easy. To deal with well-known challenges, we developed a cards-based discussion method to be used in focus groups with patients. The method aimed to enable patients (1) to put forward their experiential knowledge, (2) to develop a rich view of what an envisioned innovation might look like and do, and (3) to connect their experiential knowledge with the envisioned innovation. It is important to note that, even when successful in achieving these aims, for several reasons such a method obviously would not be a panacea for meaningful and effective patient involvement.

First of all, our project largely focused on enhancing the quality of the patients’ reflection and deliberations (and thus on the input produced by patients), rather than on improving the communication between patients and researchers, or to improve uptake of patient concerns by the researchers. This certainly limits our study, but enhancing patients’ reflections is a precondition for the added value of patient involvement in research, and poses more than enough challenges on its own.

Moreover, although the method was designed to at least partially overcome the influence of lay-expert and patient-doctor hierarchies and inequalities, as well as to mitigate differences in frames of reference that often put patients at a disadvantage, the method inevitably presupposes certain capabilities in patients (and also in moderators and participating biomedical researchers). Obviously, these may be more present in some individuals than in others, and this will mediate what the method can achieve. In addition, the method may work less well in cultures with very strong hierarchies between ‘lay people’ and ‘experts’ and patients and doctors.

Finally, we want to stress once more that our method for patient involvement, like any other alternative, is not a neutral ‘tool’. While downplaying the influence of scientific, in particular biomedical, perspectives and broadening what counts as ‘knowledge’, the method assigns a relatively large role to humanities and social scientists: as card developers, as moderators of the focus groups, and potentially as communicators of patient input to the biomedical researchers (if these are not present during the groups). We definitely tried to include a broad set of considerations in the cards, and the structuring of the conversations during the focus groups does allow patients to direct the topics of discussion, but the method unavoidably will co-shape what is being put forward by patients.

That being said, experiences with the first draft of the method showed that it was quite effective in triggering participants to voice experiences that were relevant to the technologies under discussion and in broadening imaginaries connected to this research. Participants quickly put the official motives for the research and the expectations raised by the kick-off presentation in perspective. They also were quite capable of identifying potential broader impacts of the aimed for innovation, both in patients’ lives and for society as a whole. Overall, in the discussions, many insights emerged that are pertinent to the innovation process. However, connecting these to the daily work of researchers proved more challenging. This may, first of all, have been due to the decision to phrase card texts on a rather general level. As discussed above, the moderator may have to play a more pro-active role to ensure that insights are translated into recommendations. Alternatively, the card texts could be tailored to the project under discussion. Secondly, our experiences also show that patients’ input does not always neatly match with researchers’ activities and role responsibilities. Looking at the type of concerns put forward by our participants, it seems wise to broaden the audience of the focus groups beyond researchers.

We conclude that the method *Voice of patients* can at least help to mobilise the experiential knowledge of patients for envisioned innovations. More work is needed to enable translation of patients’ considerations into the activities of researchers and others involved in work on a specific innovation, but the method we developed can be a crucial step in stimulating the development of innovations that benefit patients.
